# Structure of the Children's Revised Impact of Event Scale (CRIES) with Children and Adolescents Exposed to Debris Flood

**DOI:** 10.1371/journal.pone.0041741

**Published:** 2012-08-24

**Authors:** Zhenggen Chen, Yuqing Zhang, Zhengkui Liu, Yin Liu, Atle Dyregrov

**Affiliations:** 1 Key Laboratory of Mental Health, Institute of Psychology, Chinese Academy of Sciences, Graduate University of the Chinese Academy of Sciences, Beijing, China; 2 Center for Crisis Psychology, Bergen, Norway; The University of Queensland, Australia

## Abstract

**Aim:**

PTSD symptoms were pervasive among children and adolescents after experiencing or exposure to traumatic events. Screening and diagnosis of PTSD symptoms is crucial in trauma-related research and practice. The 13-item Children's Revised Impact of Event Scale (CRIES) has been demonstrated to be a valid and reliable tool to achieve this goal. This study was designed to examine the psychometric properties of the 13-item CRIES in a sample of Chinese debris flood victims.

**Methods:**

A total of 268 participants (145 girls, 123 boys) aged 8–18 years were recruited from an integral part of a service oriented project, supported by the Institute of Psychology, Chinese Academy of Sciences following the debris flood. The participants were given the 13-item CRIES 3 months after the debris flood.

**Results:**

The results of confirmatory factor analysis indicated that a two-factor structure (intrusion+arousal vs avoidance) emerged as the model best fit in total sample, boys and girls subsamples, respectively. The scale was also demonstrated to have good internal consistency (Cronbach's alpha = 0.83).

**Conclusion:**

The study confirmed the good psychometric properties of the CRIES and its' applicability to Chinese children and adolescents. Moreover, these findings imply that the CRIES factor structure is stable across age, gender, and different types of trauma.

## Introduction

The mental health consequences of natural disasters have been studied extensively in recent years. As a common mental health problem among survivors, posttraumatic stress disorder (PTSD) has been documented by many studies both in Western countries [1], [2], [3], [4] and Asian countries [5], [6], [7], [8], [9], [10]. Children are especially vulnerable to and may develop posttraumatic stress symptoms after experiencing a natural disaster [11], [12], [13], [14], [15]. Our recent study on the psychological effects of earthquake on children showed that PTSD symptoms were pervasive among children after experiencing earthquakes [11]. Therefore, effective screening and diagnosis of PTSD symptoms among children exposed to natural disasters is very important for mental health workers to provide effective intervention strategies to help children recover from the negative impacts of natural disasters.

The Impact of Event Scale [16], which was originally developed to assess the current degree of subjective impact of traumatic events among adult populations, has been used with children and adolescents following various traumatic events [17], [18], [19], [20], [21]. The results show that a number of items were confusing and ambiguous in children's age [17], [19]. Therefore, a revised version of the IES, the Children's Revised Impact of Event Scale (CRIES) was developed [22], with five items added in order to assess the third Cluster of PTSD symptoms, as a screening tool for probable PTSD victims among children and adolescents [24]. Since then it has become one of the most wildly used scales to screen children at risk for PTSD. However, one limitation of these researches was that they seldom reported the cross-cultural validity of the scales used in measuring PTSD [25]. Given that there are ethno-cultural differences among people's perceptions and reactions to the same disaster [26], [27], it would be enlightening to verify the cross-cultural validity of the screening instruments used for children and adolescents so as to enhance comparisons across studies in different cultures.

In an attempt to examine the cross-cultural validity of IES and CRIES, a number of studies have emerged, but empirical evidence is still scarce. Two studies found a 3-factor solution (intrusion, avoidance, emotional numbing) [28], [19] and one study reported a 2-factor solution (intrusion and avoidance) [17], [19]. among children who were exposed to either war or a shipping disaster using IES. As for CRIES, using exploratory factor analysis (EFA), Smith et al found a two-factor solution (intrusion and avoidance), with most arousal items loading onto the intrusion factor [22], in their study of 2976 children aged 9–14years who experienced war in Bosnia. However, the confirmatory factor analysis (CFA) of children exposed to the 1999 Athens earthquake produced a hierarchical three-factor solution, with intrusion, avoidance and arousal as three first-order factors and PTSD as the second-order factor [12]. Recently, we also investigated the factor structure of the CRIES as used with a large group of children who survived the 2008 Sichuan earthquake in China. In line with Giannopoulou et al. [12], a three inter-correlated factor structure, namely intrusion, avoidance and arousal, was found. These researches demonstrated that intrusion and avoidance are prominent factors of CRIES in children, while the stability and consistency of the arousal factor varied across studies. However, these studies were all conducted in Europe and only one was concerned with children in China. No empirical studies on the factor structure of CRIES have been conducted among children exposed to debris flood in China even in eastern cultures. Furthermore, earthquake and debris flooding are different types of disasters, and this may result in different posttraumatic stress reactions. Empirical research demonstrate that degree of exposure, life threat involved, and the event of physical injury correlate with reactions following trauma [29], and that “arousal factor” is likely to change its meaning when measured in different trauma situations [12].

For comparability with the research results by other researchers on the Children's Revised Impact of Event Scale [12], [24], [22] and in a continuing attempt to test the factor structure that emerged using the CRIES with children who survived the 2008 Sichuan earthquake in China [11], we conducted a confirmatory factor analysis of the scale, which was administered in a study of children and adolescents who had been exposed to the Zhouqu debris flow in China. Specifically, it was predicted that a three-factor solution (intrusion vs. arousal vs. avoidance) would fit the data better than a two-factor solution (intrusion+arousal vs avoidance). Another aim of the present study was to examine the gender and the age-group differences in factor structure, for comparability with the research results by other researchers [12], [17], [19].

## Methods

### Description of event

On August 7, 2010, about 10 p.m., a large-scale debris flow hit the Zhouqu County, in the northwest China's Gansu Province, causing extensive damage to residential and industrial buildings and public infrastructure such as schools, hospitals and transportation in the county. Two-thirds of the area of Zhouqu County was flooded and a village of 300 families was buried. Statistics based on the official reports show that 1463 deaths were reported, 302 people were reported missing, more than 2244 people were injured and more than 4496 households and 26453 houses were affected.

### Participants

One primary school and one middle school in Zhouqu County were randomly selected. One class per year level (from higher elementary school grade to primary middle school grade) was randomly selected in each school. The final study sample consisted of 268 participants (45.9% boys and 54.1% girls); 86 (32.1%) in the age range 8–11 years, 114 (42.5%) in the age range 12–14 years and 68 (25.4%) in the age range 15–18 years. The mean age of children was 12.85 years (SD = 2.19). The sample was distributed fairly evenly across the grades, with figures ranging between 14.6% and 19.4% per grade. And because Zhouqu is a county consisting of Tibet and Han people, there are 48 (18%) Tibetan and 217 (81%) Han people in our sample (3 unanswered).

### Procedure

The data was collected as an integral part of a service oriented project, in order to identify vulnerable children and to guide service planning. Approval to conduct the study was obtained from the Institutional Review Board of the Institute of Psychology, Chinese Academy of Science. Data collection was via schools 3 months after the debris flow (8th NOV 2010). Teachers and volunteers were trained to administer the CRIES-13 by class. Children completed the questionnaire anonymously in their classrooms. Oral consent was obtained both from the children and their parents before they completed the questionnaire. The participants were also assured that the data processing and presentations were completely anonymous. The reasons why written consent was not obtained from the parents were because the students had just experienced a major disaster and it was necessary to complete the survey with minimal disturbances and as fast as possible during the acute phase of post-disaster management. Also, some of the students' home were very far from their schools. They were usually living in schools and went back to home one time per month, so it was difficult to get a formal written consent from the parents under the special circumstances. The ethics committee of Institute of Psychology, Chinese Academy of Science approved this verbal consent procedure.

### Instrument

PTSD symptoms were assessed by using the 13-item Children's Revised Impact of Event Scale [22]. It was adapted from the IES-8 [23], developed as a screening instrument for children at risk of developing PTSD after experiencing a traumatic event [22]. It includes four items measuring intrusion, four items measuring avoidance and five new items measuring arousal [22]. Each item is rated on a four-point scale (Not at all, Rarely, Sometimes, Often), scored 0, 1, 3, 5 with no reversed items. The total score indicated the severity of a child's posttraumatic stress reactions with a range from 0 to 65. Earlier studies have shown that the revised scale have reasonably good psychometric properties [11], [12], [22]. Perrin et al. [24] found Cronbach's alpha to be 0.80 for the total scale and was 0.70, 0.73, and 0.60 for the intrusion, avoidance and arousal subscale, respectively. A score of 30 and above has been confirmed as the most effective cut-off score for screening cases of PTSD [24].

The Children's Revised Impact of Event Scale was translated into Chinese by Ma and So [30]. In our earlier study, a sample scale of the Chinese version of CRIES was obtained from the Children and War Foundation (http://www.childrenandwar.org/measures/) and was modified into simplified Chinese by the researchers of this project. The CRIES has demonstrated good psychometric properties in our earlier research. The internal consistency reliability of the full scale has been reported as .84 [11]. The internal consistency reliability estimates for the subscales also have been reported, ranging from the low .62 s to low .82 s [11].

### Statistical Analysis

The Statistical Package for Social Sciences (SPSS.11.5) [31] was used for assessing reliability of CRIES and subscales by means of Cronbach's alphas (internal consistency). Confirmatory factor analysis of CRIES was carried out using the Lisrel 8.7 [32].

First, the following five models were tested for the total sample. These models included: Model 1: one factor global PTSD; Model 2: two inter-correlated latent factors: (1) avoidance and (2) intrusion and arousal; Model 3: three inter-correlated latent factors: (1) intrusion, (2) avoidance and (3) arousal; Model 4: two orthogonal latent factors; Model 5: three orthogonal latent factors.

The robust maximum likelihood estimator was used in the data analysis process, and the overall goodness of each model was assessed using multiple fit indices commonly used in CFA: (1) the comparative fix index (CFI) [33], the normed fix index (NFI) [34], and the Tucker–Lewis fit index (TLI) [34], compare the fit of the hypothesized model to the independence model, and higher values are good (>0.90, acceptable, and >0.95, desirable; [35]); (2) the root mean square error of approximation (RMSEA; [36]), which is an index of the fit between hypothesized model and the saturated model; lower values are preferred (<0.08, acceptable, <0.06 desirable; [35]); (3) the standardized root-mean-square residual (SRMR; [33]), which is an index of the average standardized deviation between the model-based reproduced covariances in contrast to those observed in the data; lower values are desirable (<0.08; [34]); (4) the Goodness of Fit Index (GFI) and the Adjusted Goodness of Fit Index (AGFI), where values of above 0.9 denote acceptable model fits [34]; (5) the Bayesian information criterion (BIC; [37]), which is useful in comparing nonnested models, and a difference of 6–10 indicates strong support and a difference greater than 10 indicated very strong support for the model with lower BIC value [38]. The BIC is not included in LISREL 8.7 output and was thus calculated separately using the following formula: BIC = +ln(N)×t, where N = sample size and t = number of parameters estimated in the model.

## Results

### Internal consistency

For the CRIES, Cronbach's alpha for all items was 0.83. Regarding the three different subscales, Cronbach's alpha for the intrusion and avoidance items was 0.74 and 0.72 respectively, whereas lower internal consistency was found for the five arousal items, with Cronbach's alpha at 0.59.

### Posttraumatic stress symptoms

Mean score on the CRIES-13 was 27.84 (SD = 12.40) for the total sample, 27.59(SD = 13.02) for boys, and 28.05(SD = 11.88) for girls. No significant difference was found between boys and girls in terms of the total scores and the scores on the three subscales,nor between the Tibetan and the Han ethnics (Table 1). Based on previous research [24], a ‘probable PTSD case’ was identified by using a cut-off of a total score of 30 on the CRIES-13. According to this criterion, 125 (46.6%) participants were identified as probable PTSD cases and out of which 59 (40.7%) were boys, and 66 (53.7%) were girls.

**Table 1:** Sample characteristics and Group differences on the CRIES and its subscales.

		N(%)	intrusion	avoidance	arousal	Total
Gender	boys	123(45.9)	8.29±4.94	8.80±5.47	10.50±5.24	27.59±13.02
	girls	145(54.1)	8.39±4.35	8.70±5.10	10.96±5.03	28.05±11.88
Ethnicity	Tibetan	48(17.9)	8.39±4.73	8.60±4.91	9.96±5.00	27.00±11.52
	Han	217(81)	8.34±4.64	8.77±5.38	10.91±5.18	28.02±12.68

Notes: CRIES = Children's Revised Impact of Event Scale.

Total = Children's Revised Impact of Event Scale Total Score.

**Figure 1:**
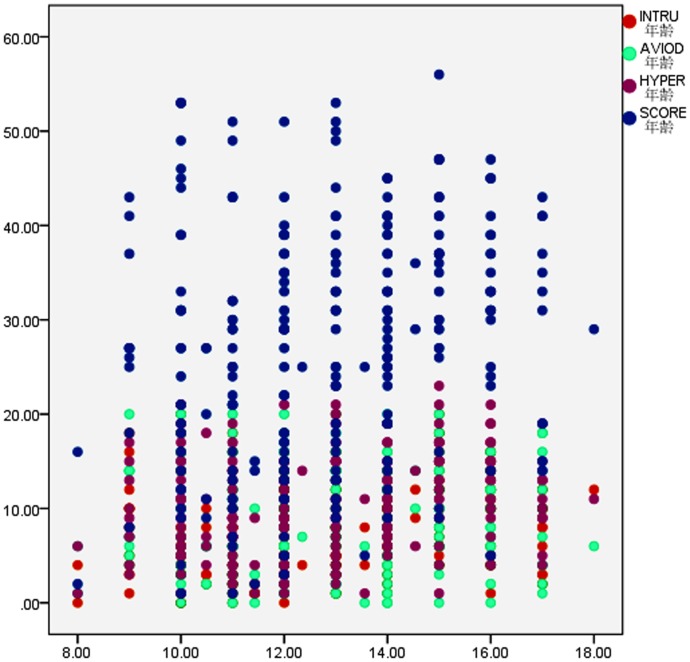
The scatter plot of regression analysis between CRIES score and age. Notes: INTRU = intrusion; AVIOD = avoidance; HYPER = arousal; SCORE = Children's. Revised Impact of Event Scale Total Score.

However, an interesting finding was that older children scored higher than younger on the total response score and on the two subscales, except the avoidance factor (see Table 2 and Figure 1).

**Table 2:** Regression Analysis for age predicting the score on the CRIES and its subscales.

		B	SE B	β
Age	intrusion	0.43	0.13	0.21**
	avoidance	0.22	0.15	0.09
	arousal	0.67	0.14	0.29**
	total	1.33	0.34	0.24**

Notes: CRIES = Children's Revised Impact of Event Scale.

Total = Children's Revised Impact of Event Scale Total Score.

** p<0.01;

* p<0.05.

### Factor structure of the CRIES

Fit statistics for the hypothesized models for the complete data set (N = 268) are summarized in Table 3. According to the criteria mentioned above, only Model 1 and Model 5 did not achieve acceptable fit. Furthermore, the indices unanimously suggested the superiority of the inter-correlated models over the orthogonally rotated factor models. This provides further evidence that the factors of CRIES are highly correlated with each other rather than independent of each other. However, compared with the three inter-correlated factor model, the two inter-correlated factor model had smaller BIC-value, and BIC differences (all above 10) provided very strong support for this model. Therefore, the two inter-correlated factor model was chosen as the best fitting model.

**Table 3:** Goodness-of-fit indices for the specified models across the whole sample (N = 268).

model	X^2^	df	CFI	TLI	RMSEA(95% confidence interval)	SRMR	AIC	BIC
One factor global PTSD	228.57	65	0.93	0.91	0.097(0.084–0.11)	0.071	280.57	373.936
Two factors	106.15	64	0.98	0.97	0.050(0.032–0.066)	0.055	160.15	257.107
Three factors	105.83	62	0.97	0.97	0.051(0.034–0.068)	0.055	163.83	267.969
Two-factor orthogonal	159.81	65	0.94	0.92	0.074(0.060–0.088)	0.150	211.81	310.767
Three-factor orthogonal	326.83	65	0.84	0.81	0.12(0.11–0.14)	0.220	378.83	488.969

Note. CFI = Comparative fit index; TLI = Tucker-Lewis index; RMSEA = Root mean square error of approximation; SRMR = standardized root mean square residual AIC = Akaike information criterion. BIC = Bayesian information criterion.

After establishing the underlying factor structure on the basis of the entire sample, we tested whether the same factor model held for different subgroups of the data set. For this purpose, the data set was divided into boys and girls, as well as into two different age groups (group 1: 8–12 years, group 2: 13–18 years old). The inter-correlated two-factor model was then fitted separately to (1) boys and girls, and (2) across the different age groups. As shown in Table 4, the specified model provided an adequate fit for both sex groups, as well as for the different age groups. All fit indices exceeded the set thresholds and the RMSEA indicated a good approximation fit.

**Table 4:** Goodness-of-fit indices for the two-factor model fitted separately to the different gender and age groups.

model	X^2^	df	CFI	TLI	RMSEA (95% confidence interval)	SRMR	AIC	BIC
Boys 2	64.59	64	1.00	1.00	0.0087(0.0–0.055)	0.053	118.59	194.52
Boys 3	62.67	62	1.00	1.00	0.0094(0.0–0.056)	0.052	120.67	202.22
Girls 2	105.16	64	0.94	0.93	0.067(0.043–0.089)	0.076	159.16	239.53
Girls 3	104.38	62	0.94	0.92	0.069(0.045–0.091)	0.076	162.38	248.71
8–12 2	93.92	64	0.97	0.96	0.062(0.032–0.088)	0.073	147.92	223.85
8–12 3	91.31	62	0.97	0.96	0.062(0.032–0.088)	0.072	149.31	230.86
13–18 2	87.59	64	0.97	0.96	0.051(0.018–0.075)	0.066	141.59	221.96
13–18 3	87.34	62	0.97	0.96	0.053(0.023–0.078)	0.066	145.34	231.67

Note. CFI = Comparative fit index; TLI = Tucker-Lewis index; RMSEA = Root mean square error of approximation; SRMR = standardized root mean square residual AIC = Akaike information criterion. BIC = Bayesian information criterion.

## Discussion

As in our previous study [11], which was aimed to evaluate the psychometric properties of the CRIES and its applicability in China by using it to assess traumatic stress in children and adolescents who survived the 2008 Sichuan earthquake, this further study provides further support for the scale among different individuals following a different type of traumatic event - debris flow. The internal consistency (Cronbach's alpha) of the total PTSD score was satisfactory, with acceptable values for the three subscales, consistent with previous research [12], [22]. According to the response scores on CRIES, the result showed that 46.6% of the children and adolescents survivors of the Zhouqu debris flow were likely to develop posttraumatic stress symptoms. This result is congruent with our earlier studies on rates of PTSD among children and adolescents exposed to Sichuan earthquake [11]. It is also in line with other studies on rates of PTSD among children and adolescents exposed to earthquakes [39], [40], [15], [41]. The differences among different age groups' scores on CRIES-total and the subscales showed that older children scored higher than younger on the total response score and on the two subscales. It was in accordance with previous research which showed that older children suffered more reexperiencing and arousal after a traumatic event [42], [37], [43]. One possible explanation for this difference is that age and developmental level may influence the exposure to risk and the perception and understanding of trauma, susceptibility to parental distress, coping style and skills, memory of the event, adaptation, social skills, and self-concept. However, there was no difference on avoidance symptoms. This may reflect that avoidance symptoms are common in children and adolescents because avoiding bad things is the nature of human beings. Further studies are needed to clarify the mechanisms of this phenomenon.

We also investigated the factor structure of the CRIES in a sample of children and adolescents from China who recently experienced the large-scale debris flood. The results of CFA indicated that the two inter-correlated factor model (avoidance; intrusion and arousal) emerged as the model that had the best fit for the total sample, in the boys and girls sub-sample, and in the different age groups. Contrary to predictions, our three-factor structure (intrusion, arousal, avoidance) found in Sichuan earthquake was not replicated. This may not be caused by sample bias, because these two studies have similar samples, like gender, age and ethnic groups. In the earlier study, 49.6% boys and 50.4% girls for the first sample, and 44.9% boys and 55.1% girls for the second sample, with the mean age of 13.96 (SD = 1.36; range from 10–17) years and 11.75 (SD = 1.61; range from 10–18) years respectively. Thus,this may due to the fact that children in these two studies experienced the different types of disasters. In the previous study, most children and adolescents were directly exposed to the devastating earthquake when it hit during school hours; while a majority of this Zhouqu sample didn't experience the debris personally, they just witnessed the scene after the disaster. Therefore, for this indirect exposure sample, intrusion and arousal factors maybe so highly correlated that they were combined into one factor. This is in line with Keppel-Benson & Ollendick's study [29], which demonstrated that degree of exposure, life threat involved, and the event of physical injury correlate with reactions following trauma. It is also consistent with that of Giannopoulou et al. [12], who found “arousal factor” is likely to change its meaning when measured in different trauma situations. Another possible explanation is that there is indeed a strong overlap between intrusion and arousal symptoms. In the factor analytic study of the CRIES-13, Smith et al. [22] found that the arousal items loaded very highly on the 4-item intrusion scale. Furthermore, it is notable that in the other previous study, intrusion and arousal are indeed more highly correlated than either intrusion and avoidance, or arousal and avoidance [12], [22]. Future research should attempt to examine the stability of the arousal factor of CRIES following different types of traumatic events involving different types of exposure.

The present study confirms earlier findings that intrusion and avoidance are robust and separable factors of the CRIES in different cultural and different types of trauma situation, It suggests that post-traumatic stress reactions transcend cultural barriers and that the CRIES factor structure is robust across different types of trauma. As there were no differences in factor structure in different age groups, or between genders, it indicated that children and adolescents appear to have the same set of underlying mechanisms for the stress response reactions, such as intrusion and avoidance, if each factor represents a distinct set of mechanisms [44]. Therefore, our study provided further support that children from diverse cultures are more similar than different in the ways they react to traumatic events. This may be helpful when designing transnational mental health assistance for trauma affected children following disasters.

Together with the earlier study, this study confirmed the good psychometric properties of the CRIES and its' applicable among Chinese children and adolescents this time with a different type of natural disaster. However, the factor structure of the CRIES found in this sample was different from the earlier study. Consistent with Perrin et al. [24] we found that the arousal items did not stand out as a separate factor by themselves and most of them loaded on the intrusion factor. One possible explanation is the different type traumatic events experienced by the children. Another is that a strong overlap between intrusion and arousal symptoms does indeed exist. Taken brevity into consideration, further research should evaluate the performance of the CRIES 8 as a screening tool following disasters specially. As has been pointed out elsewhere [45], [46], screening instruments for PTSD are better when they contain the minimum number of items necessary to accurately identify individuals with the disorder. Additional research examining the third cluster of arousal items is needed and underway. Future research should attempt to examine the stability of the arousal factor of CRIES following different types of traumatic events involving different types of exposure.

There are several limitations to this study. First, Self-report instruments were used; hence, the responses may have been over- or under- reported. Second, because of the sample limitations and the different time after exposure to the disaster of the two studies, it's not an ideal design to compare these two studies. Last, our study focused on debris flood survivors in China. The results cannot necessarily be generalized to other types of disaster or to debris flood survivors in other countries. Notwithstanding these limitations, the present study provided further empirical evidence that CRIES-13 can be used as a screening instrument for probable PTSD among children and adolescents after natural disasters such as debris flood. And the two inter-correlated factor model (avoidance; intrusion and arousal) emerged as the model best fit in total sample, boys and girls subsamples is consistent with findings from studies of other disasters and cultures [17], [22]. This also implies that post-traumatic stress reactions are not culture-bound, and that the CRIES factor structure is stable across age, gender, and different types of trauma.
